# Complete mitochondrial genome of the Korean oily shinner *Sarcocheilichthys nigripinnis morii* (Cypriniformes, cyprinidae): mitogenome characterization and phylogenetic analysis

**DOI:** 10.1080/23802359.2016.1247674

**Published:** 2016-11-11

**Authors:** Seungki Lee, Ha Yeun Song

**Affiliations:** a Biological and Genetic Resources Assessment Division, National Institute of Biological Resources, Incheon, Republic of Korea;; b Genetic Resources Team, National Marine Biodiversity Institute of Korea, Seocheon-gun, Republic of Korea

**Keywords:** Cypriniformes, cyprinidae, gobioninae, *Sarcocheilichthys nigripinnis morii*, Korean oily shinner

## Abstract

The Korean oily shinner (*Sarcocheilichthys nigripinnis morii*) is a subspecies belonging to the subfamily Gobioninae in the family Cyprinidae and is endemic to Korea. Herein, we report the first sequencing and assembly of the complete mitochondrial genome of *S. nigripinnis morii*. The complete mitochondrial genome is 16,679 bp long, consisting of 13 protein-coding genes, 22 tRNA genes, 2 rRNA genes, and a control region. It has the typical vertebrate mitochondrial gene arrangement. Phylogenetic analysis using mitogenomes of 16 species showed that *S. nigripinnis morii* was clustered with *S. nigripinnis* and *S. variegatus microoculus* and grouped with the other Cyprinidae species.

The Korean oily shinner, *Sarcocheilichthys nigripinnis morii* (Jordan & Hubbs 1925), is a subspecies belonging to the subfamily Gobioninae in the family Cyprinidae and is endemic to Korea. This subspecies is allopatrically distributed in upstream and midstream regions in southwestern Korea (Kim & Park 2007). To date, there has been very little molecular and genetic research on this species. To the best of our knowledge, this is the first study to determine the complete mitochondrial genome of *S. nigripinnis morii* and to analyze the phylogenetic relationship of this subspecies among Cypriniformes fishes.

The *S. nigripinnis morii* specimen (standard length, 93.6 mm) was collected from the Mangyeong River, South Korea (35.47°N, 127.11°E). The specimen was deposited in the National Institute of Biological Resources (NIBR, Voucher No. NIBRGR0000098804). Genomic DNA from muscle tissue was sequenced and assembled using the Illumina Hiseq 4000 sequencing platform (Illumina, San Diego, CA) and *SOAPdenovo* assembler at Macrogen Inc. (Korea), respectively. The complete mitochondrial genome was annotated using MacClade ver. 4.08 (Maddison & Maddison 2005) and DNASIS ver. 3.2 (Hitachi Software Engineering). Experiments were conducted in accordance with the Guidelines of Animal Ethics published by the NIBR.

The complete mitochondrial genome of *S. nigripinnis morii* (GenBank accession no. AP017653) is 16,679 bp long and includes 13 protein-coding, 22 tRNA, and 2 rRNA genes. The *ND6* gene and eight tRNA genes are encoded on the light strand. The overall base composition of the heavy strand is 29.83% A, 26.70% C, 17.21% G, and 26.24% T. As in mitogenomes of other vertebrates (Saccone et al. 1999), the AT content is higher than the GC content. All tRNA genes can fold into a typical cloverleaf structure and are 68–76 bp long. The 12S rRNA (958 bp) and 16S rRNA genes (1690 bp) are located between tRNA^Phe^ and tRNA^Val^ and between rRNA^Val^ and tRNA^Leu(UUR)^, respectively. Except for *COI*, which starts with GTG, the 12 protein-coding genes start with ATG. Seven of these terminate with incomplete stop codons, T (*COII, COIII,* and *Cytb*) and TA (*ND2, ATP6, ND3*, and *ND4*), whereas the remaining six genes ended with complete stop codons (TAA or TAG). A control region (1,007 bp) is located between tRNA^Pro^ and tRNA^Phe^.

Phylogenetic trees were constructed (maximum likelihood) with 1000 replicates using MEGA 7.0 software (PA, USA) (Kumar et al. 2016) for the newly sequenced mitogenome and a further 15 complete mitogenome sequences downloaded from the National Center for Biotechnology Information. We confirmed that *S. nigripinnis morii* is clustered with *S. nigripinnis* (Tang et al. 2013) and *S. variegatus microoculus* (Saitoh et al. 2003) and grouped with the other Cyprinidae species (Figure 1). Because *S. nigripinnis morii* exhibits a unique spawning symbiosis with mussels such as *Anodonta arcaeformis* (Kim & Park 2007; An et al. 2016), it is vulnerable to habitat degradation. We initiated a project to conserve them through the use of interspecies spermatogonial transplantation (Lee et al. 2013; Lee et al. 2015). This mitogenome provides a potentially important resource for developing conservation strategies and addressing taxonomic issues.

**Figure 1.**
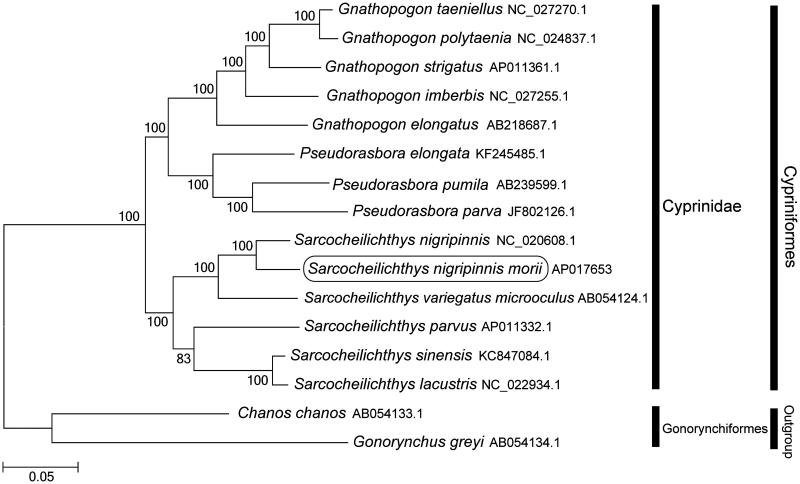
Phylogenetic position of *Sarcocheilichthys nigripinnis morii* based on a comparison with the complete mitochondrial genome sequences of 13 Cyprinidae species and 2 outgroup species. The analysis was performed using the MEGA 7.0 software. The accession numbers for each species are indicated after the scientific names.

## References

[CIT0001] An C, Ouyang S, Zhou CH, Wu XP. 2016 The complete F-type mitochondrial genome of Chinese *Anodonta arcaeformis* (Bivalvia: Unionidae: Anodontinae). Mitochondrial DNA A DNA Mapp Seq Anal. 27:1552–1553. 25208165 10.3109/19401736.2014.953133

[CIT0002] Jordan DS, Hubbs CL. 1925 Record of fishes obtained by David Starr Jordan in Japan. Mem Carneg Mus. 10:93–346.

[CIT0003] Kim IS, Park JY. 2007 Freshwater fishes of Korea. Seoul, Korea: Kyo-Hak Publishing.

[CIT0004] Kumar S, Stecher G, Tamura K. 2016 MEGA7: molecular evolutionary genetics analysis version 7.0 for bigger datasets. Mol Biol Evol. 33:1870–1874. 27004904 10.1093/molbev/msw054 PMC8210823

[CIT0005] Lee S, Iwasaki Y, Shikina S, Yoshizaki G. 2013 Generation of functional eggs and sperm from cryopreserved whole testes. Proc Natl Acad Sci USA. 110:1640–1645. 23319620 10.1073/pnas.1218468110 PMC3562789

[CIT0006] Lee S, Seki S, Katayama N, Yoshizaki G. 2015 Production of viable trout offspring derived from frozen whole fish. Sci Rep. 5:16045. 26522018 10.1038/srep16045 PMC4629203

[CIT0007] Maddison DR, Maddison WP. 2005 MacClade 4: analysis of phylogeny and character evolution. ver. 4.08. Sunderland (MA): Sinauer Associates.

[CIT0008] Saccone C, De Giorgi C, Gissi C, Pesole G, Reyes A. 1999 Evolutionary genomics in metazoa: the mitochondrial DNA as a model system. Gene. 238:195–209. 10570997 10.1016/s0378-1119(99)00270-x

[CIT0009] Saitoh K, Miya M, Inoue JG, Ishiguro NB, Nishida M. 2003 Mitochondrial genomics of ostariophysan fishes: perspectives on phylogeny and biogeography. J Mol Evol. 56:464–472. 12664166 10.1007/s00239-002-2417-y

[CIT0010] Tang WR, Lin HD, Su LW, Tang WQ, Wu CY, Yang JQ. 2013 The complete mitochondrial genome sequence of *Sarcocheilichthys nigripinnis* (Cypriniformes, Cyprinidae). Mitochondrial DNA. 24:478–480. 23438052 10.3109/19401736.2013.770496

